# Annotation of genes involved in high level of dihydromyricetin production in vine tea (*Ampelopsis grossedentata*) by transcriptome analysis

**DOI:** 10.1186/s12870-020-2324-7

**Published:** 2020-03-30

**Authors:** Xiaohua Li, Minhui Cao, Weibo Ma, Caihua Jia, Jinghuan Li, Mingxing Zhang, Changchun Liu, Zhenzhen Cao, Mohammad Omar Faruque, Xuebo Hu

**Affiliations:** 1 Laboratory of Natural Medicine and Molecular Engineering, Department of Medicinal Plant, College of Plant Science and Technology, Huazhong Agriculture University, Wuhan, Hubei China; 2 Laboratory of Drug Discovery and Molecular Engineering, Department of Medicinal Plants, College of Plant Science and Technology, Huazhong Agricultural University, Wuhan, 430070 China; 3 National-Regional Joint Engineering Research Center in Hubei for Medicinal Plant Breeding and Cultivation; Medicinal Plant Engineering Research Center of Hubei Province, Huazhong Agricultural University, Wuhan, 430070 China; 4 Department of Chemistry, College of Science, Huazhong Agriculture University, Wuhan, Hubei China; 5 Key Laboratory of Environment Correlative Dietology (Ministry of Education), College of Food Science and Technology, Huazhong Agricultural University, Wuhan, Hubei China

**Keywords:** Vine tea, Transcriptome analysis, Phenylpropanoid pathway, Gene expression, Dihydromyricetin

## Abstract

**Background:**

Leaves of the medicinal plant *Ampelopsis grossedentata*, which is commonly known as vine tea, are used widely in the traditional Chinese beverage in southwest China. The leaves contain a large amount of dihydromyricetin, a compound with various biological activities. However, the transcript profiles involved in its biosynthetic pathway in this plant are unknown.

**Results:**

We conducted a transcriptome analysis of both young and old leaves of the vine tea plant using Illumina sequencing. Of the transcriptome datasets, a total of 52.47 million and 47.25 million clean reads were obtained from young and old leaves, respectively. Among 471,658 transcripts and 177,422 genes generated, 7768 differentially expressed genes were identified in leaves at these two stages of development. The phenylpropanoid biosynthetic pathway of vine tea was investigated according to the transcriptome profiling analysis. Most of the genes encoding phenylpropanoid biosynthesis enzymes were identified and found to be differentially expressed in different tissues and leaf stages of vine tea and also greatly contributed to the biosynthesis of dihydromyricetin in vine tea.

**Conclusions:**

To the best of our knowledge, this is the first formal study to explore the transcriptome of *A. grossedentata.* The study provides an insight into the expression patterns and differential distribution of genes related to dihydromyricetin biosynthesis in vine tea. The information may pave the way to metabolically engineering plants with higher flavonoid content.

## Background

Vine tea (Teng cha) is a famous traditional Chinese tea, made from leaves of *Ampelopsis grossedentata* (Hand.-Mazz.) W.T. Wang [1]. The plant is widely distributed in mountainous areas of southern China and is frequently consumed [1]. Vine tea has been considered to be a functional, healthy beverage for hundreds of years in China. Unlike the conventional green tea (*Camellia sinensis* L.), which contains high levels of catechins [2, 3], the main bioactive metabolite of vine tea is dihydromyricetin (DHM), a flavanonol compound [4]. DHM, also known as ampelopsin, plays important roles in various pharmacological activities such as antioxidant [1], anti-inflammatory [5, 6], antimicrobial [7], reno-protective [8], and anti-cancer activities [9, 10], etc. [11]. In addition, DHM plays a therapeutic role in the treatment of ischemic stroke [12]. Vine tea has the capability to inhibit melanogenesis and is also used in depigmentation skin care products [13].

Secondary metabolites play an essential role in plant defense system and development processes [14–16]. The accumulation of secondary metabolites can be influenced by environmental factors and different plant growth stages [17–20]. Previous studies have reported that the metabolites of green tea (*C. sinensis*) varied according to geography, developmental stages, and cultivars [2, 21, 22]. Another study reported that the distribution of major phenolic compounds is dominant in younger rather than older plant parts [23]. The quality of green tea mainly depends on the catechin content in leaves. However, the content of catechins usually varies with leaf stage, altitude, and season [2, 3]. Moreover, several studies have illustrated various biosynthetic patterns for metabolites in *C. sinensis*, especially at the transcriptional level, using an Illumina HiSeq transcriptome sequencing technique [24–26].

Phenylpropanoids are a large group of compounds that play a vital role as secondary metabolites in vine tea. The structural genes in the phenylpropanoid biosynthetic pathway have been well studied [27–30]. In recent years, with the development of the Illumina HiSeq technique, the transcriptomic analysis has successfully been applied in a wide range of species to explore the secondary metabolic pathway [25, 31–33]. However, the mechanisms of the phenylpropanoid biosynthetic pathway in vine tea remains unclear. In the present study, we applied transcriptome sequencing and conducted profiling analysis of vine tea to provide a clear understanding of the phenylpropanoid biosynthetic pathway.

## Results

### High-performance liquid chromatography analysis of the DHM content in vine tea

**Fig. 1:**
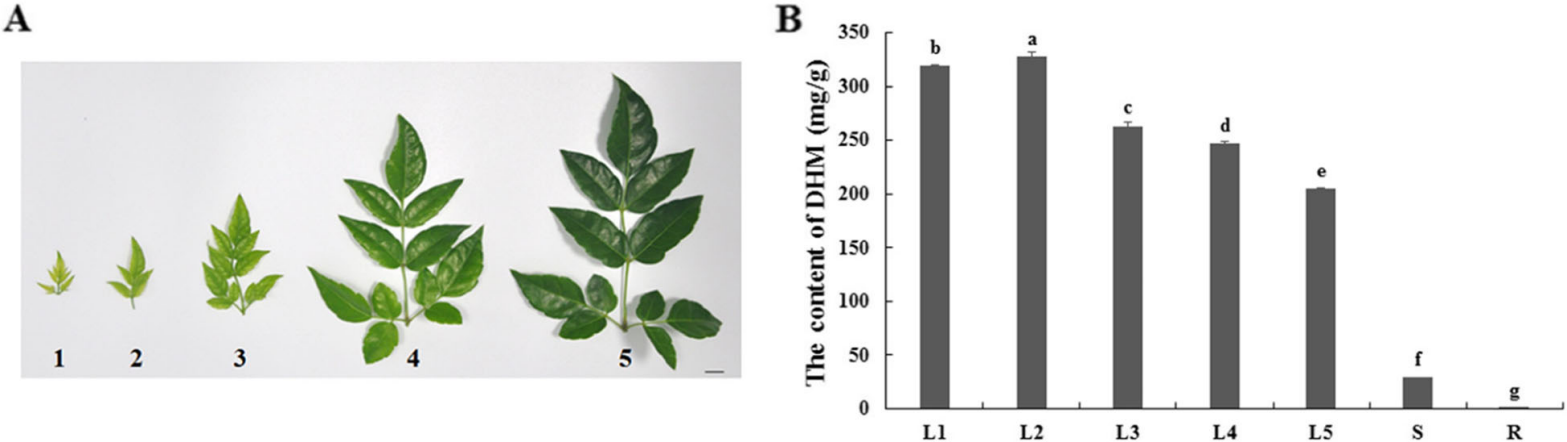
The content of dihydromyricetin (DHM) in different leaf stages and tissues of *A. grossedentata*. **a**. The young and old leaves from *A. grossedentata*. 1–3: young leaves; 4–5: old leaves. Leaves part were used for experiment without branch, bar indicate 1 cm. **b**. The content of dihydromyricetin (DHM) in different tissues of vine tea. L1–3: young leaves; L4–5: old leaves, S: stems, R: roots. The height of each bar represents a mean of 3 replicates, ANOVA was processed for statistical analysis by using SPSS 18.0 software

The DHM content was quantified in different leaf stages and tissues of vine tea by high-performance liquid chromatography (HPLC; Fig. 1, Additional file 1: Figure S1). Vine tea leaves were divided into five growth stages (Fig. 1a). A higher amount of DHM was detected in developing young leaves (Stage 1: 319.23 mg/g, Stage 2: 327.84 mg/g, and Stage 3: 263.25 mg/g, respectively) as compared to old leaves (Stage 4: 247.32 mg/g, and Stage 5: 204.55 mg/g) of vine tea (Fig. 1b). The highest level was determined in young leaves at stage 2, which is one stage beyond that of the apical point. In different tissues of vine tea, significantly lower content of DHM was recorded in stems (29.50 mg/g) and roots (0.27 mg/g) (Fig. 1b). These notable differences in content indicated the varying accumulation of DHM in different leaf growth stages and tissues of vine tea.

### Transcriptome sequencing and de novo assembly

We sequenced cDNA libraries prepared from the young and old leaves using an Illumina HISeq 2000 to characterize the transcriptome of *A. grossedentata*. The transcriptome reads were assembled using Trinity [34]. Sequencing of the young and old leaves of *A. grossedentata* (three samples of each) subsequently generated 157.4 million and 141.8 million reads in total and 52.47 and 47.25 million reads on average, respectively, after removing adapter containing low-quality reads (Additional file 2: Table S1). The maximum and minimum read lengths were 101 and 50 bases, respectively. The quality of raw reads was determined using SeqQC-V2.2. Short read values were compiled using assembly software, which improved the quality of the transcriptome assembly.

The transcriptome sequencing was performed using three independent biological replicates. A summary of the Hi-Seq Illumina transcriptome assembly is presented in Table 1. Out of 471,658 transcripts, 351,096 transcripts (74.44%) were 200–500 bp in length, 63,451 transcripts (13.45%) were 500 bp–1000 bp long, and 57,111 transcripts (12.11%) were longer than 1000 bp (Table 1). In both samples, the average length of the contigs ranged from 200 bp to more than 1000 bp. The de novo reads were assembled into a gene transcriptome dataset with an N50 length of 1633 bp and N90 length of 469 bp (Additional file 3: Table S2). All raw sequences were uploaded to the NCBI Sequence Read Archive (accession number PRJNA608806).

**Table 1:** The length distribution of unigenes and transcripts from the transcriptome data of *A. grossedentata*

Transcript length interval	200-500 bp	500-1kbp	1 k-2kbp	>2kbp	Total
Number of transcripts	351,096	63,451	32,795	24,316	471,658
Number of Genes	61,555	58,817	32,734	24,316	177,422

### Gene functional annotation and classification of *A. grossedentata* transcriptome

A total of 177,422 unigenes were annotated in seven public databases namely the NCBI non-redundant protein sequences database (Nr), NCBI non-redundant nucleotide database (Nt), Protein family database (Pfam), euKaryotic Orthologous Groups (KOG), Gene Ontology (GO), Clusters of Orthologous Groups of protein databases (COG) and Swiss-Prot protein database (Table 2). The number of genes annotated by the five representative databases (Nt, Nr, Kog, Go, and Pfam) is presented in a Venn diagram (Additional file 4: Figure S2). In total, 21,556 unigenes (12.14%) were commonly aligned to the homologous sequences before being mentioned in open databases, while 152,254 unigenes (85.81%) were annotated in at least one open database (Table 2). E-value distribution and the similarity distribution of the best blast hits against the NCBI Nr database for unigenes are shown in Additional file 5: Figure S3. In addition, the species homologous distribution analysis of unigenes is shown as the percentage of the total homologous sequences according to the Nr database. Altogether, 8229 unigenes (59.9%) were highly matched with the sequences obtained from *Vitis vinifera*, followed by *Coccomyxa subellipsoidea* (4.8%), *Hordeum vulgare* (2.9%), *Picea sitchensis* (2387, 2.0%), and *Galdieria sulphuraria* (1117, 0.9%).

**Table 2:** Summary statistics of functional annotations for unigenes from *A. grossedentata* via public databases

	Number of Unigenes	Percentage (%)
Annotated in NR	98,164	55.32
Annotated in NT	121,715	68.6
Annotated in KO	41,091	23.16
Annotated in SwissProt	78,039	43.98
Annotated in PFAM	75,984	42.82
Annotated in GO	78,265	44.11
Annotated in KOG	47,612	26.83
Annotated in all databases	21,556	12.14
Annotated in at least one database	152,254	85.81
Total Unigenes	177,422	100

Gene Ontology (GO) enrichment analysis showed that the unigenes were annotated into 56 sub-categories. The highest enriched categories were ‘cellular process’, ‘metabolic process’, ‘binding’ and ‘catalytic activity’, which belong to the ‘biological process’ and ‘molecular function’ categories in vine tea (Additional file 6: Figure S4). According to the KOG classification system and Kyoto Encyclopedia of Genes and Genomes (KEGG), unigenes were divided into 26 KOGs and 19 pathways, respectively (Additional files 7 and 8: Figure S5–6). A major proportion of unigenes (47,612) was found to be complementary to *A. grossedentata* and showed homology to general function prediction, translation, ribosomal structure, biogenesis and posttranslational modification, protein turnover, and chaperone genes (Additional file 7: Figure S5). Accordingly, the highest number of unigenes (6542) showed homology to the cluster of translation cluster, followed by carbohydrate metabolism (4600) and biosynthesis of secondary metabolites (1066) (Additional file 8: Figure S6).

### Analysis of differentially expressed genes (DEGs)

**Fig. 2:**
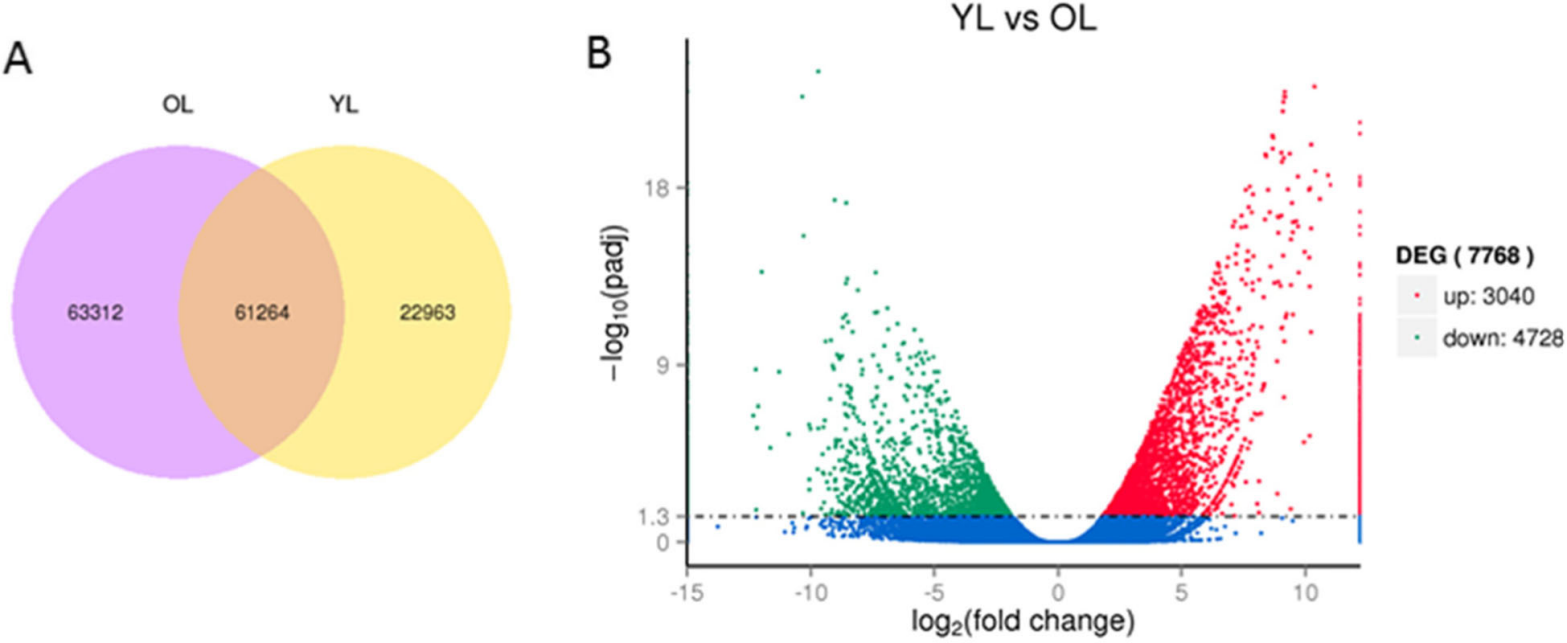
The differentially expressed genes (DEGs) between the young and old leaves in vine tea. **a**. Venn diagram of differentially expressed genes in vine tea; **b**. YL vs OL volcano plot. (|log2. Fold_change| > 1, *q* value< 0.005)

**Fig. 3:**
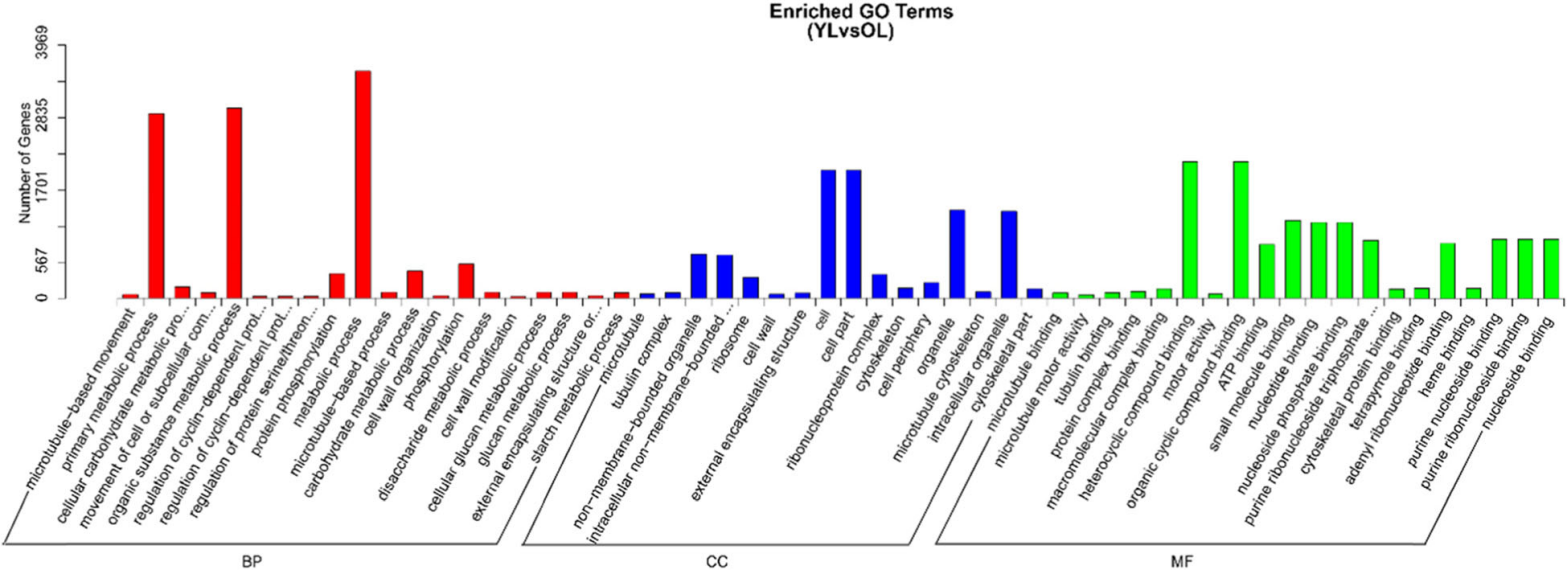
The enriched GO classification of differentially expressed genes (DEGs) from the young and old leaves of vine tea. BP, biological process; CC, Cellular component; MF, molecular function

**Fig. 4:**
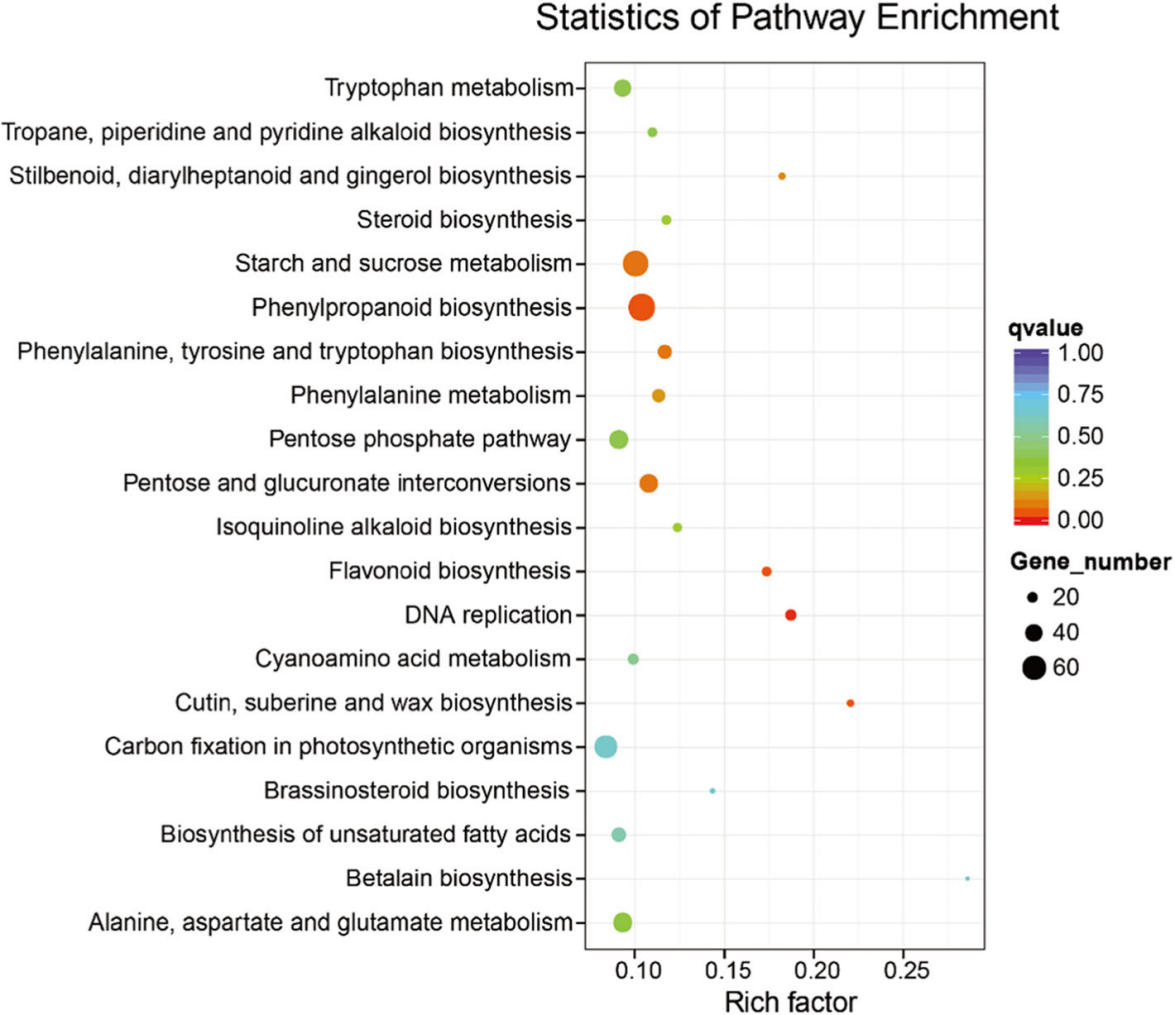
Scatter plot of YL vs OL DEGs enriched KEGG pathway. YL: young leaves; OL: old leaves

Differentially expressed genes in young leaves and old leaves of vine tea plants were analyzed to compare the transcriptome expression patterns. Our results showed that, out of 177,422 expressed genes, 61,264 unigenes were found in both young and old leaves, while 7768 genes were differentially expressed between the two leaf stages (Fig. 2a). We also found that a large number of transcripts (4728) were significantly down-regulated in young leaves compared with old leaves (Fig. 2b). According to the GO classification of DEGs, the remainder of the genes showed homology with genes for metabolic process, organic substance metabolic process and primary metabolic process (Fig. 3). KEGG pathway enrichment analysis showed that these genes differentially expressed between young and old leaves were involved mainly in phenylpropanoid biosynthesis and starch and sucrose metabolism (Fig. 4, Additional file 9: Table S3).

### Phenylpropanoid biosynthetic pathway gene expression in vine tea

**Fig. 5:**
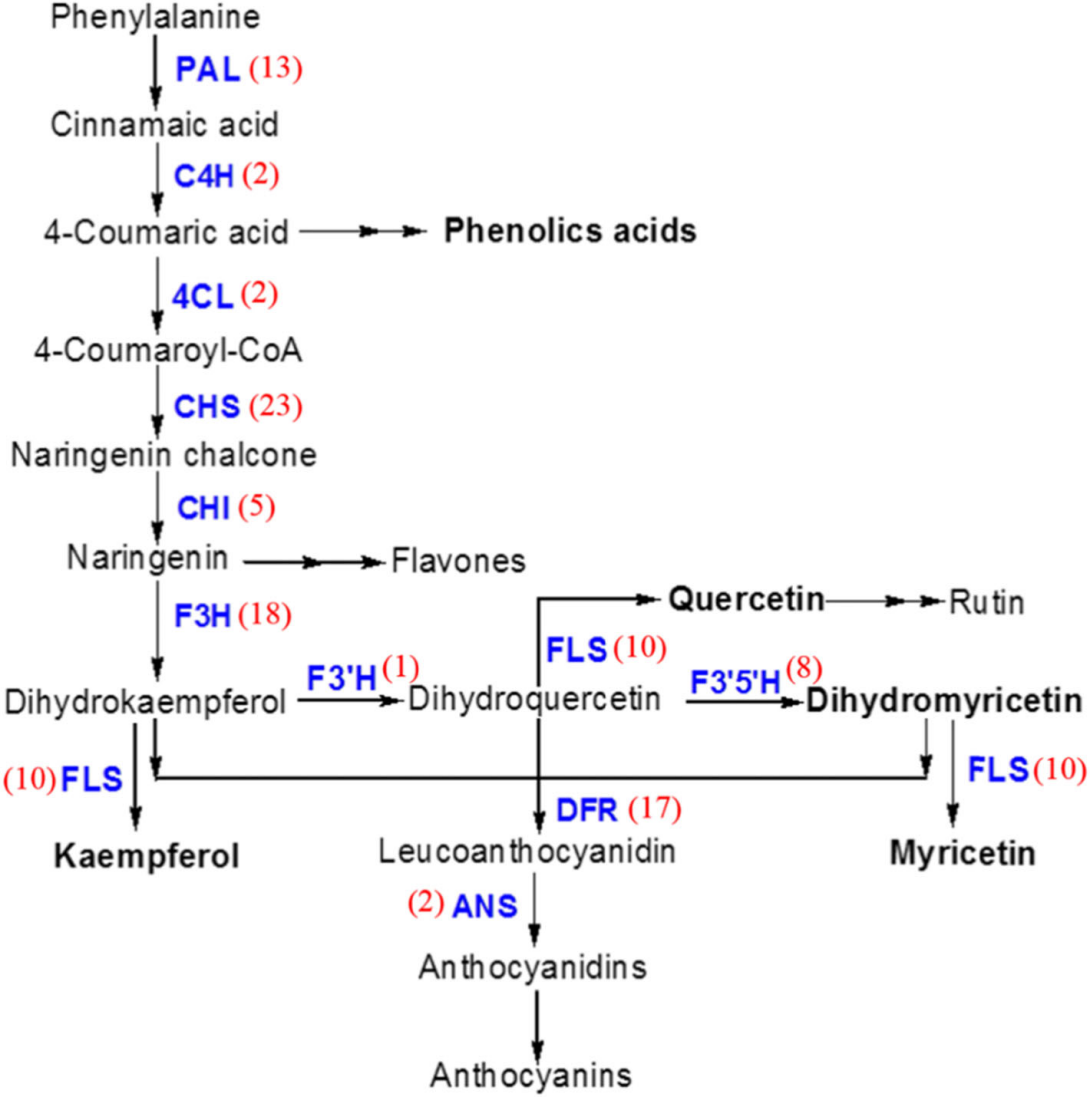
Phenylpropanoid biosynthetic pathways in *A. grossedentata*. The main phenylpropanoid biosynthetic pathways genes were marked in blue color. The numbers in brackets indicated the unigene identified by RNA-Seq. PAL, phenylalanine ammonium lyase; C4H, cinnamic acid 4-hydroxylase; 4CL, 4-coumarate-CoA ligase; CHS, chalcone synthase; CHI, chalcone isomerase; F3H, flavanone-3-hydroxylase; F3′H, flavonoid-3′-hydroxylase; F3’5’H, flavonoid-3′,5′-hydroxylase; FLS, flavonol synthase; DFR, dihydroflavonol reductase; ANS, anthocyanin synthase

**Fig. 6:**
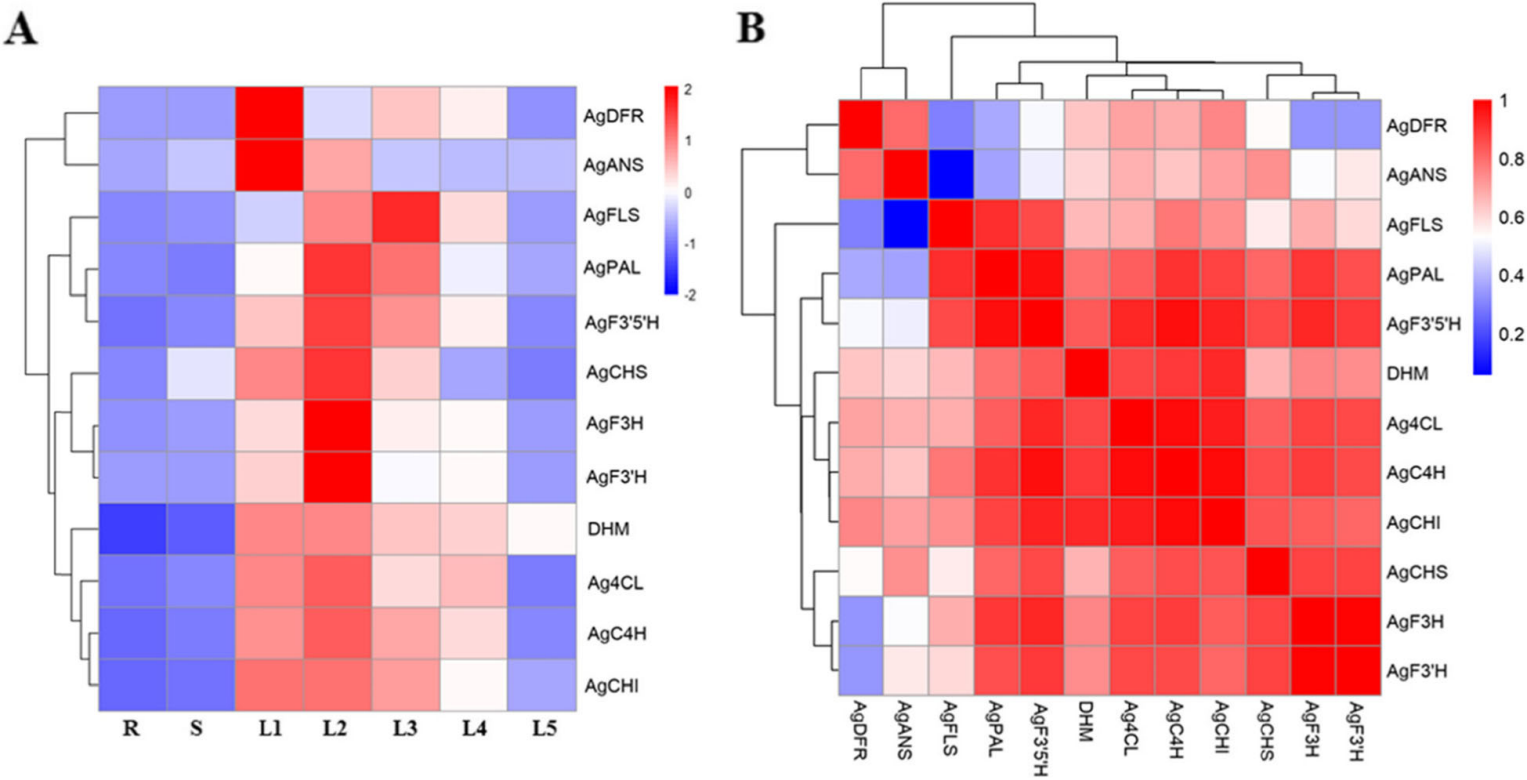
The heatmap of genes expression (**a**) and the correlation analysis between the accumulation of DHM and the expression of biosynthesis related genes (**b**) in different leaf stages and tissues of vine tea. R: roots, S: stems, L1-L3: young leaves, L4- L5: old leaves. Pearson correlation coefficients were calculated and used to generate a heatmap (B), Each square indicates the Pearson’s correlation coefficient of a pair of data, and the value for the correlation coefficient is represented by the intensity of the blue or red color, as indicated on the color scale

Most of the known transcripts associated with the phenylpropanoid biosynthesis pathway were found more abundantly in young leaves than old leaves. Based on the KEGG database, a total of 124 genes and isoforms were annotated in the phenylpropanoid biosynthetic pathway (Table 3 and Additional file 10: Table S4). For corroboration of the annotated transcripts and to evaluate the differential gene expression profile between leaves at various stages, some transcripts cognate to those for phenylpropenoid biosynthesis were selected for qRT-PCR analysis (Figs. 5 and 6). We found that most of the transcripts related to the phenylpropanoid pathway, including genes encoding cinnamic acid 4-hydroxylase (C4H), 4-coumarate-CoA ligase (4CL), chalcone synthase (CHS), chalcone isomerase (CHI), flavanone-3-hydroxylase (F3H), flavonoid-3′-hydroxylase (F3’H) and flavonoid-3′,5′-hydroxylase (F3’5’H), were more highly expressed in young leaves than old leaves (Fig. 6a). The significantly differential gene expression profile was also detected in tissues from different organs of vine tea. Obviously, most of the main phenylpropenoid biosynthesis structural genes exhibited lower levels of expression in roots, stems and old leaf stage 5. Exceptionally, the *AgANS* gene showed a stable, lower expression level in leaf stages 3–5 and tissues of vine tea (Fig. 6a). A correlation analysis of the main structural genes of the phenylpropenoid biosynthetic pathway and DHM was carried out (Fig. 6b). The results indicated that a significantly positive correlation was observed between DHM and most of the genes in the phenylpropanoid biosynthetic pathway. (Fig. 6b).

**Table 3:**
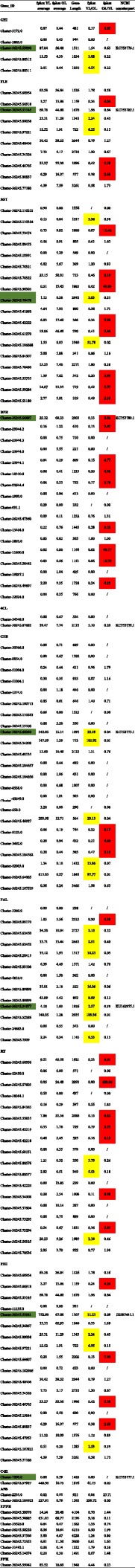
Transcript abundance of phenylpropanoid biosynthetic pathways genes identified in *A. grossedentata* transcriptome

The genes and transcripts dealing phenylpropanoid biosynthetic pathway in *A. grossedentata* were identified. The average fpkm for young (YL) and old (OL) leaves from 3 samples each, the gene length and the ratio were shown. Fpkm ratio of YL/OL and OL/YL was given wherever the value greater than 2 was marked in yellow and red, respectively. The key pathway genes, whose counterparts were also found in NCBI database, highlighted with green, were used as template for q-PCR in Fig. 6

## Discussion

Vine tea is a frequently used herbal tea in south China. The raw material of vine tea is processed by one-step natural fermentation [35] and the product is consumed generally as a tonic, because it contains a high level of the flavonoid DHM [1, 36]. Crude extracts of vine tea plants demonstrate high antioxidant activity compared to tertiary butylhydroquinone [36]. Previous studies have disclosed that the quality of green tea (*C. sinensis* L.) varies according to the seasons and plant developmental stages [37, 38]. A similar phenomenon has been noticed in vine tea plants during this study.

Previous researchers have reported that the contents of DHM and other flavonoids may vary within different plant parts, developmental stages and seasonal conditions in vine tea plants [39, 40]. Equally, based on geographical environment, the main chemical components of vine tea vary; for example, the contents of DHM and myricetin vary between 0.53–33.58% and 0–0.48%, respectively, in the southern areas of China [41]. Although a few samples have yielded low DHM contents (< 1%), in most samples the DHM content is relatively high with a range of 18.03 –33.58% [36, 41]. Some previous studies have shown that leaf age can reduce the quality of vine tea [39, 40]. In this study, HPLC data confirmed the differential accumulation of the DHM in different tissues and various leaf stages of vine tea (Fig. 1). The DHM content recorded in the old leaves corroborates with results of previous studies [36, 41]; however, stage 2 young leaves yielded 1.3–1.6 times more of the compound than older leaves at stages 4–5 old leaves, and DHM content of stems and roots were rarely detected in vine tea. Commonly, both the old and young shoots are harvested together for the commercial production of vine tea; however, the young shoots are usually graded as having better quality and being worth a higher price. With differences between different tissues of vine tea at the transcriptional and chemical level, our results revealed information useful to further agricultural practice and mechanistic study. The quality of vine tea might be graded by age or even the chemical content of the leaves of vine tea. This observation is relevant in terms of green tea quality. Traditionally, the best quality green tea is obtained from the newly developed buds. One study demonstrated that the theanine and anthocyanins in the buds are present at significantly higher levels than those in other leaves of green tea plans [42]. The bud and three young leaves next to the bud also have higher percentages of catechins than the older leaves [42]. The results of the present study demonstrate that the DHM content is higher in the younger shoots than older leaves or other tissues of vine tea. However, it is still unclear whether the higher DHM content in the shoots is transferred from older leaves or it is locally synthesized in the shoots.

The annotation of non-model species by transcriptome analysis is a common strategy for exploring a unique profile. Since it is rare for any chemical compound to make up greater than 10% of a plant part by weight, it is significant that DHM alone makes up nearly one-third of the components of vine tea leaves and over 40% of young leaves by weight. As there are no related data available, we conducted a whole genome transcript analysis to shed light on the biosynthesis of DHM and related flavonoid metabolites in the plant. Using RNAs from the young and old leaves, we performed detailed transcriptome analysis of vine tea and found 7768 differentially expressed unigenes. The best matches for individual unigene searches against the NCBI non-redundant database and KEGG were promoted to assign functional GO annotation under different biological processes. After mapping the unigenes onto KEGG, we found that most of them are involved in phenylpropanoid biosynthesis and starch and sucrose metabolism. The GO classification analysis also revealed that the differentially expressed genes were highly involved in organic substance and primary metabolic processes. The results reveal that the transcripts encoded a large number of genes involved in biosynthesis of flavonoids specifically found in young leaves of vine tea.

Out of 138 genes and isoforms found to be associated with the flavonoid biosynthetic pathway, there were 5 for CHI, 10 for FLS, 18 for 3GT, 17 for DFR, 23 for CHS, 13 for PAL, 18 for RT, 18 for F3H, 8 for F3’5’H, 2 each for 4CL, C4H and ANS, and 1 each for C4H and F3’H (Fig. 5). Putting aside possible inaccuracies brought about by different lengths of sequence assembly, the gene numbers are 2.7 times greater than those for green tea, which has 51 unigenes for catechin, a compound with a structure similar to DHM [25]. Only one unigene, PAL, in the green tea plant has over 10 isoforms, while the rest have one to three isoforms [25]. It is highly likely that vine tea has a tendency to contain more unigenes or isoforms for each of these genes. The FPKM indicates a statistical relationship with the gene expression level. However, the ratios of each gene in the young and old leaves appear to be quite random, with some unigenes showing higher numbers in young leaves and some higher in old leaves (Table 3). It is perplexing that some genes such as *AgCHI*, *AgFLS*, *Ag3GT*, *AgCHS*, *AgPAL*, *AgRT*, and *AgF3H* all have different unigenes with high levels of expression in both young and old leaves. Only one gene, *DFR*, demonstrated a consistent higher FPKM in old leaves (Table 3). This discrepancy will be investigated further using functional analysis in a future study.

The expression levels of some genes in the flavonoid biosynthesis pathway were examined using quantitative RT-PCR analysis (Fig. 6). All primers used in this study were designed based on previous published data [42] and that from the NCBI. Only one fragment was chosen for each gene and the counterparts for each of the genes can be matched to one of our RNA-Seq unigenes (Table 3). The q-PCR result showed that of 11 fragments tested, most of them exhibited much stronger expression in the young leaves than old leaves, indicating that the biosynthetic activity in the young leaves is possibly more active than in the old leaves. The young leaves of vine tea showed substantially higher transcript levels of *AgC4H*, *Ag4CL*, *AgCHS*, *AgCHI*, *AgF3H*, *AgF3’H*, and *AgF3’5’H* genes compared with old leaves, stems, and roots. The correlation analysis indicated that significantly positive correlation existed between DHM and the main structural genes including *AgC4H*, *Ag4CL*, *AgCHI*, *AgF3H*, *AgF3’H*, and *AgF3’5’H* (Fig. 6). There was lower correlation between DHM and *AgFLS*, *AgANS*, and *AgDFR*, which were not directly involved in DHM biosynthesis. Our findings indicated that the higher expression levels of genes related to phenylpropenoid biosynthesis might contribute the higher accumulation of DHM in young leaves rather than old leaves or other tissues in vine tea. However, the true unigene numbers should be further verified by molecular cloning and functional analysis. Further experiments may also help to determine the behavior of these unigenes in young leaves and old leaves.

In addition, it has been demonstrated that many transcription factors (TFs), including MYBs, NACs, basic helix–loop–helix (bHLH), WD40s, and WRKYs, are involved in the regulation of metabolic biosynthesis in plants and the regulatory pattern of these transcription factors is usually tissue-specific [43–47]. MYBs play a critical role in fruit and flower color formation via the regulation of structural genes in the flavonoid or anthocyanin biosynthetic pathway [43–45, 48–51]. It has been reported that R2R3 MYB transcription factors act as important regulators in controlling the spatiotemporal biosynthesis of flavonoids during grape and apple fruit development [44, 51]. Two MYB transcription factors, *CsMYB2* and *CsMYB26*, were reported to be involved in flavonoid biosynthesis in tea (*C. sinensis* (L.) O. Kuntze) [52]. Additionally, *GmMYB58* and *GmMYB205* were demonstrated to be seed-specific activators for isoflavonoid biosynthesis in *Glycine max* [45]. Numerous reports have indicated that transcription factors from MYB, bHLH, and WD-repeat (WDR) families generally form a ternary complex (MBW complex), which coordinately activates or represses multiple genes in the regulatory network to influence metabolic biosynthesis [46, 47, 53, 54]. Our study has identified a number of transcription factors including MYB, bHLH, and bZIP genes from vine tea (Additional file 11: Table S5). Of the 4825 transcription factors identified, the MYBs, C2H2s, bHLHs, and bZIPs were significantly differentially regulated between young and old leaves. In total, 34 and 16 MYB genes, 4 and 49 C2H2s, 20 and 12 bHLHs, and 9 and 22 bZIPs were up- or down-regulated between young and old leaves of vine tea, respectively (Additional file 11: Table S5), which may play an important role in regulating DHM biosynthesis in vine tea plant. Understanding these transcription factors at the molecular level will be significant to raising flavonoid levels in vine tea.

## Conclusions

In this study, a comparative transcriptome analysis of young and old leaf tissues of *A. grossedentata* was performed to explore the putative transcripts involved in the flavonoid biosynthetic pathway of this important tea plant. The differential expression pattern of genes observed in leaves at different stages of development and in different tissues of vine tea suggests tissue-specific synthesis and accumulation of flavonoid metabolites, particularly DHM. This study provides a useful resource for investigating the flavonoid biosynthetic pathway to determine the reasons behind the significant accumulation of and number of transcripts identified for certain flavonoids in vine tea. The information may pave a way to metabolically engineer this plant with increased flavonoid content.

## Methods

### Plant materials and culture conditions

Vine tea (*A. grossedentata*) plants were grown on a private farm in Laifeng County, Enshi Area, Hubei Province, China (29°14′N, 109°26′E). The tea was grown in a natural environment. Leaves at different growth stages and tissues of vine tea were collected in the month of June, 2016. The plants were identified using a species identification key by Professor Dr. Xuebo Hu [55], College of Plant Science and Technology, Huazhong Agricultural University, Wuhan, China. A voucher specimen was deposited at a local herbarium, the Herbarium of Huazhong Agricultural University (Branch CCAU of Chinese Virtual Herbarium), with accession numbers HBES0801 and HBES0802. Leaves at five growth stages were separated from apical buds to old branches for HPLC analysis (Fig. 1). Samples were rapidly frozen in liquid nitrogen after collection and stored at − 80 °C until analysis. Samples were also prepared and sent to Novogene Company (Beijing, China) for cDNA library construction and transcriptome analysis.

### RNA isolation, library preparation, and transcriptome sequencing

The samples of three repeats were pooled and ground into powder in a mortar using liquid nitrogen. Total RNA was extracted from around 100 mg of powder using a Trizol reagent (Invitrogen). The quality of mRNA including purity, quantity, and integrity was tested using Nanodrop spectrophotometer (IMPLEN, CA, USA), Qubit RNA Assay Kit (Life Technologies, CA, USA), and Agilent Bioanalyzer 2100 system (Agilent Technologies, CA, USA). A total amount of 1.5 μg RNA per sample was used as input material for the RNA sample preparations. Sequencing libraries were generated using NEBNext® Ultra™ RNA Library Prep Kit for Illumina® (NEB, USA) following manufacturer’s recommendations and index codes were added to attribute sequences to each sample. Finally, the purified double-stranded cDNA samples were further enriched by PCR to construct the final cDNA libraries that were sequenced using Hiseq 2500 (150 bp paired ends) by Novogene Company (Beijing, China).

### Total RNA extraction and cDNA synthesis

The total RNA was isolated from *A. grossedentata* using an RNeasy Plant Mini Kit (Qiagen, Valencia, CA, USA) TIANGEN RNAprep Pure kit (TIANGEN). For first-strand cDNA synthesis, one μg of high-quality total RNA was used for reverse transcription (RT) with a Takara FastQuant RT Kits (Takara, Japan). A 20-fold dilution of 20 μL of the resulting cDNA was used as a template for quantitative real-time PCR.

### Quantification real-time PCR (qRT-PCR) analysis

The transcript levels of different samples from *A. grossedentata* were analyzed by qRT-PCR analysis. The gene-specific primers were designed to perform qRT-PCR analysis for some major transcripts involved in flavonoid biosynthetic pathway (Fig. 5, Additional file 12: Tab. S6). Here the housekeeping gene *GAPDH* from *A. grossedentata* was used as an internal standard [56]. Gene expression was normalized to that of the housekeeping gene *GAPDH*. Real-time PCR reactions were performed in triplicate on a MiniOpticon system (Bio-Rad Laboratories, Hercules, CA, USA) using the SYBR Premix Ex Taq™ (TIANGEN, China). Each run contained a series of standards and negative control (using water instead of cDNA). The qRT-PCR protocol was as follows: denaturation at 95 °C for 5 min, followed by 40 cycles of denaturation at 95 °C for 15 s, annealing at 56 °C for 15 s, and elongation at 72 °C for 20 s. The triplicate was conducted for each sample, and the qRT-PCR results were calculated as the mean of 3 replicated treatments.

### Extraction of flavonoids from vine tea

The samples of vine tea were freeze-dried and ground into powder. Flavonoid extraction was performed as followed. Samples were ground to fine powder, and 0.1 g powdered material was extracted with 1.5 mL of 70% methanol (MeOH). The extraction material was vortexed for 1 min followed by sonication for 0.5 h at 30 °C. After centrifugation at 1000×*g* for 5 min, the supernatant was filtered through a 0.45-μm PTFE syringe filter (Cameo 25F, Micron Separations Inc., Westboro, MA) and used for metabolites analysis.

### Quantitative HPLC analysis

The HPLC analysis of flavonoids was performed on a Waters HPLC system (Milford, MA, USA) equipped with a waters ODS column (150 mm × 4.6 mm, five μm; Waters Corp., Milford, MA, USA). The mobile phase consisted of a mixture of (A): acetic acid-water (0.15%) and (B) MeOH. The initial mobile phase composition was 20% solvent B, followed by a linear gradient from 5 to 80% of solvent B. Detection was performed at 285 nm, and the column oven temperature was 30 °C. The flow rate was set at 0.8 mL/min, and the injection volume was ten μL. (+)-Dihydromyricetin (DHM) standard compounds (> 99%) were purchased from Sigma Chemicals Co. (St. Louis, MO). Quantification of the different compounds was calculated as equivalents of representative standard compounds. All contents were expressed as mg/g of dry weight.

### Data processing and statistical analysis

Data shown in figures was expressed as the mean of 3 independent replicates. Experimental data were processed by analysis of variance (ANOVA), and significant differences among the means were determined by Duncan’s multiple-range test (SPSS 22.0, Chicago: SPSS Inc.). Values of *p* < 0.05 were considered statistically significant. Pearson correlation analysis was calculated by SPSS 22.0, and the correlation coefficient was calculated using the means of metabolite concentrations and genes relative expression values. Then cluster analysis and heatmap were drawn by R (http://www.r-project.org/).

## Supplementary information

**Additional file 1: Figure S1.** The HPLC chromatograms of DHM analysis in young and old leaves of vine tea. A, The standards chromatograms of dihydromyricetin (1), rutin (2), myricetin (3), quercetin (4) and kaempferol (5) at 285 nm; B and C, HPLC chromatograms of young (B) and old (C) leaves of vine tea.

**Additional file 2: Table S1.** Reads annotated with reference database.

**Additional file 3: Table S2.** The distribution of transcripts and genes length based on De novo assembly.

**Additional file 4: Figure S2.** Annotation of all the unigenes from vine tea transcriptome. Number of genes annotated by five representatively databases (nt, nr, kog, go, pfam) was showed in Venn diagram.

**Additional file 5: Figure S3.** Characteristics of unigenes annotated against Nr databases. A, E-value distribution of the top BLAST hits for each unigene (E-value of 1.0e^− 5^); B, Similarity distribution of the top BLAST hits for each unigene; C, homologous species distribution against Nr database.

**Additional file 6: Figure S4.** Gene Ontology (GO) classification of unigenes of *A. grossedentata*.

**Additional file 7: Figure S5.** KOG Function classification the transcriptome of *A. grossedentata*.

**Additional file 8: Figure S6.** KEGG classification of unigenes of *A. grossedentata*.

**Additional file 9 Table S3.** YL VS OL DEG enriched KEGG Pathway top 20.

**Additional file 10: Table S4.** Transcript abundance of phenylpropanoid biosynthetic pathways genes identified in *A. grossedentata* transcriptome.

**Additional file 11: Table S5.** The differentially expressed transcription factors (TFs) in vine tea.

**Additional file 12: Table S6.** Nucleotide sequences of primers used for qRT-PCR.

## Data Availability

The transcriptome clean raw reads data in this study have been deposited in NCBI Sequence Read Archive (https://www.ncbi.nlm.nih.gov/Traces/sra_sub/) with the accession number PRJNA608806. The relevant data in this study are included in this article and the supplementary files.

## Abbreviations

4CL: 4-coumarate-CoA ligase
ANS: Anthocyanin synthase
C4H: Cinnamic acid 4-hydroxylase
CHI: Chalcone isomerase
CHS: Chalcone synthase
DEGs: Differentially expressed genes
DFR: Dihydroflavonol reductase
*F3*’5’*H*: Flavonoid-3′,5′-hydroxylase
F3′H: Flavonoid-3′-hydroxylase
F3H: Flavanone-3-hydroxylase
FLS: Flavonol synthase
GO: Gene ontology
HPLC: High-performance liquid chromatography
KEGG: Kyoto Encyclopedia of Genes and Genomes
KOG: Eukaryotic Ortholog Groups
Nr: NCBI non-redundant protein sequences
Nt: NCBI non-redundant nucleotide sequences
PAL: Phenylalanine ammonium lyase
