# Supplementary figures and images for: Annotation of genes involved in high level of dihydromyricetin production in vine tea (Ampelopsis grossedentata) by transcriptome analysis
Source: BMC Plant Biol. 2020 Mar 30;20:131. doi: 10.1186/s12870-020-2324-7 (PMC7106717; doi:10.1186/s12870-020-2324-7)

## Slide 1
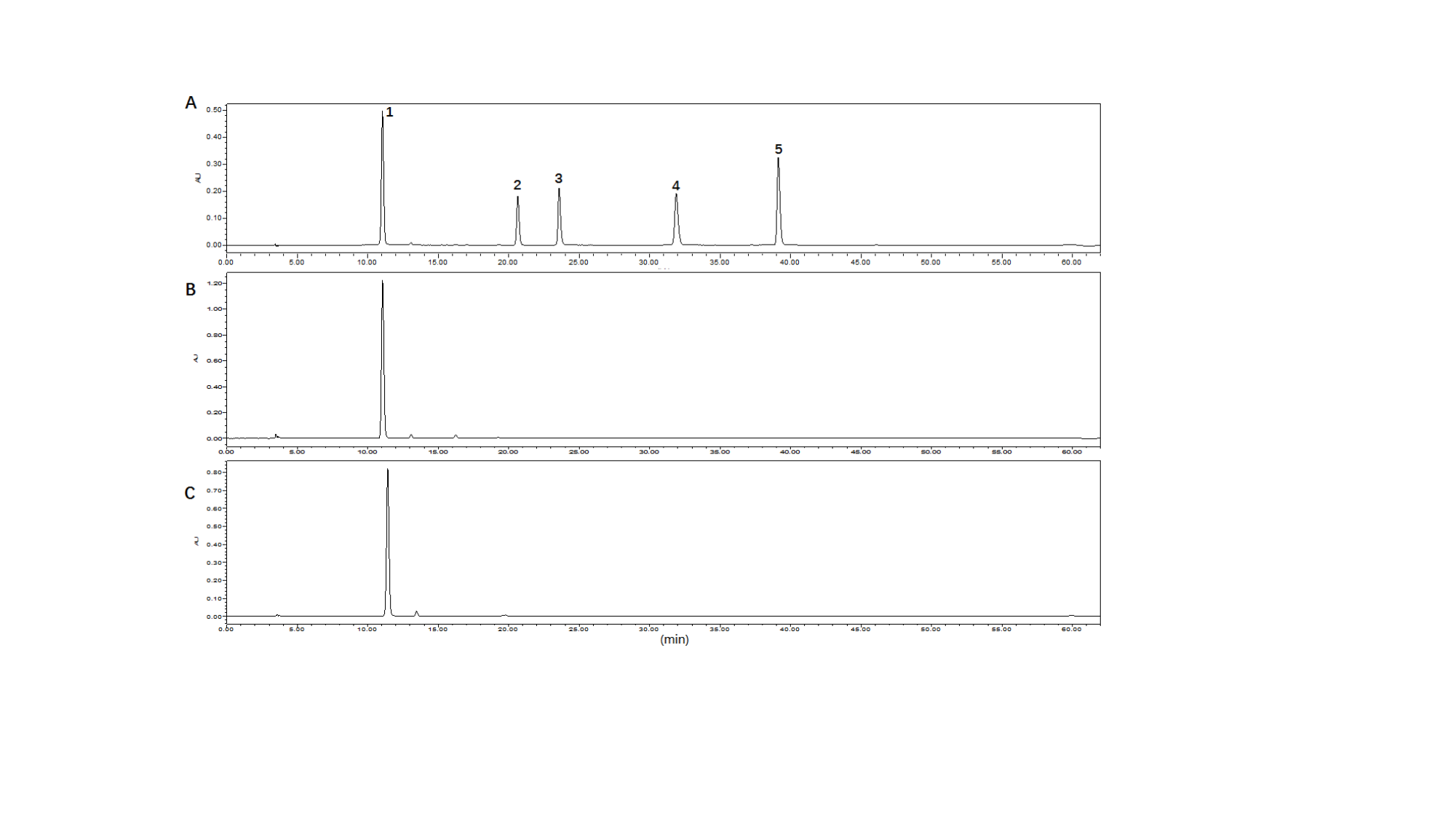

Supplement: Supplementary file 1 — Additional file 1: Figure S1. The HPLC chromatograms of DHM analysis in young and old leaves of vine tea. A, The standards chromatograms of dihydromyricetin (1), rutin (2), myricetin (3), quercetin (4) and kaempferol (5) at 285 nm; B and C, HPLC chromatograms of young (B) and old (C) leaves of vine tea. [file 12870_2020_2324_MOESM1_ESM.pptx]

## Slide 1
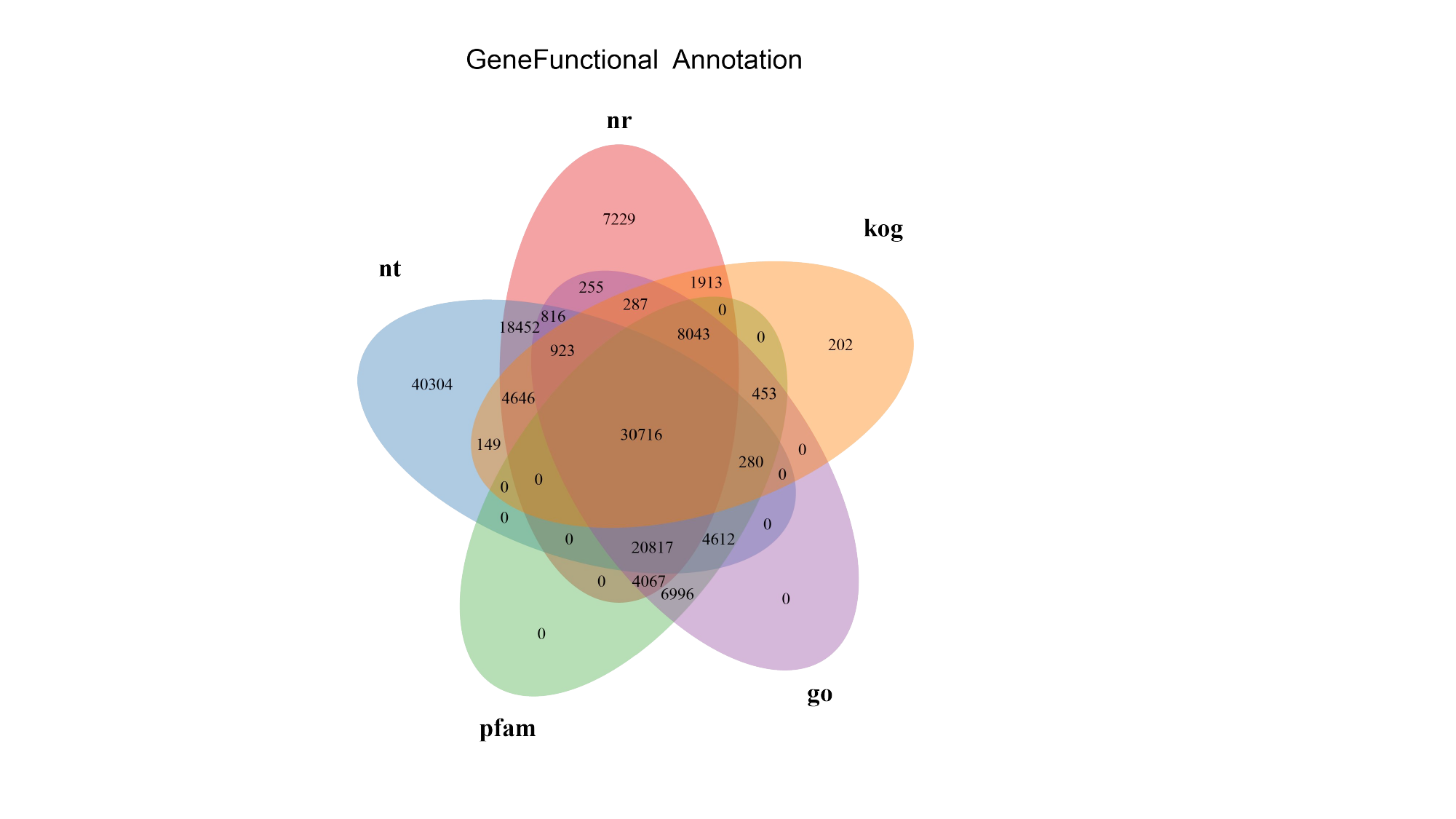

Supplement: Supplementary file 4 — Additional file 4: Figure S2. Annotation of all the unigenes from vine tea transcriptome. Number of genes annotated by five representatively databases (nt, nr, kog, go, pfam) was showed in Venn diagram. [file 12870_2020_2324_MOESM4_ESM.pptx]

## Slide 1
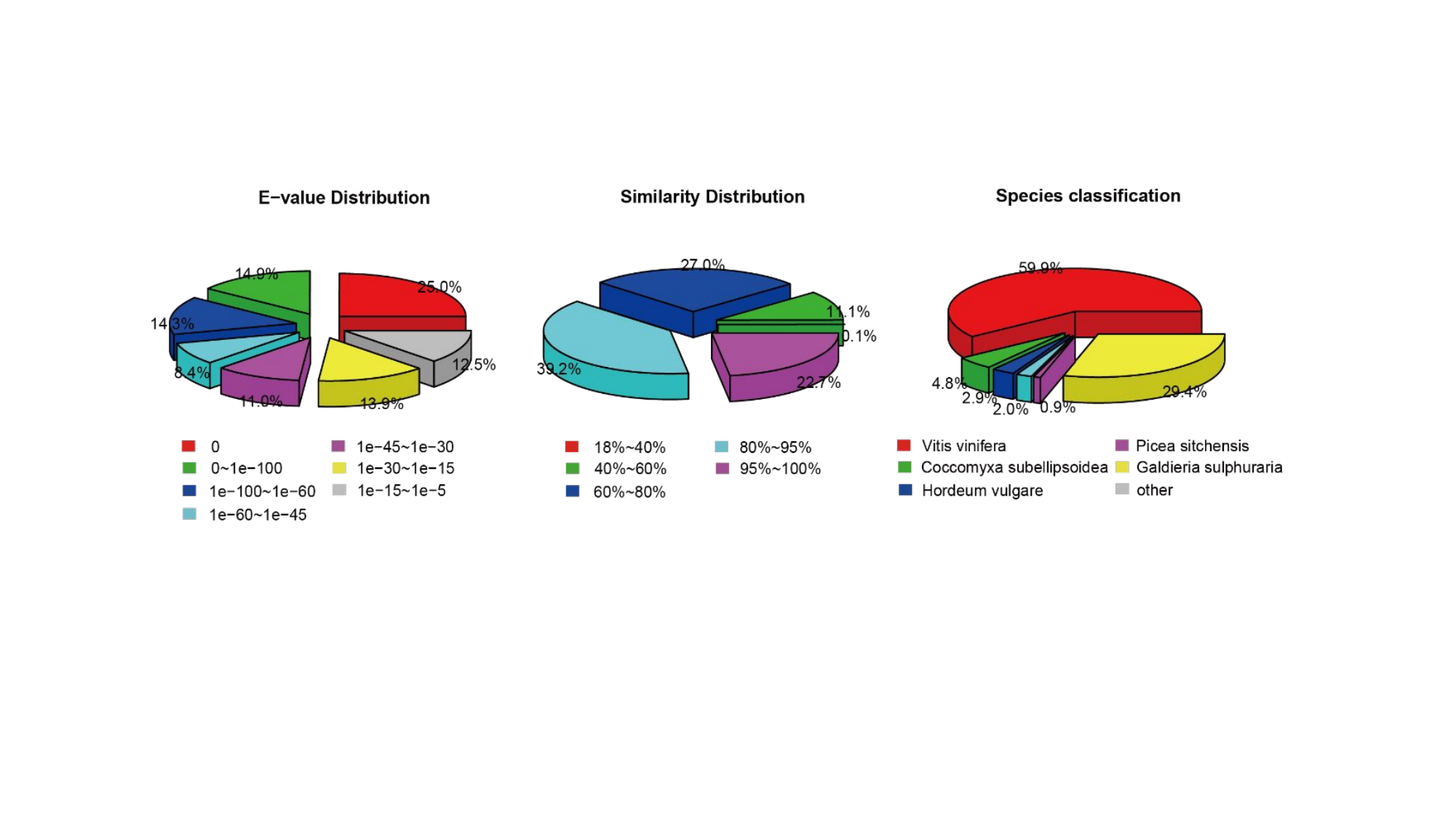

Supplement: Supplementary file 5 — Additional file 5: Figure S3. Characteristics of unigenes annotated against Nr databases. A, E-value distribution of the top BLAST hits for each unigene (E-value of 1.0e− 5); B, Similarity distribution of the top BLAST hits for each unigene; C, homologous species distribution against Nr database. [file 12870_2020_2324_MOESM5_ESM.pptx]

## Slide 1
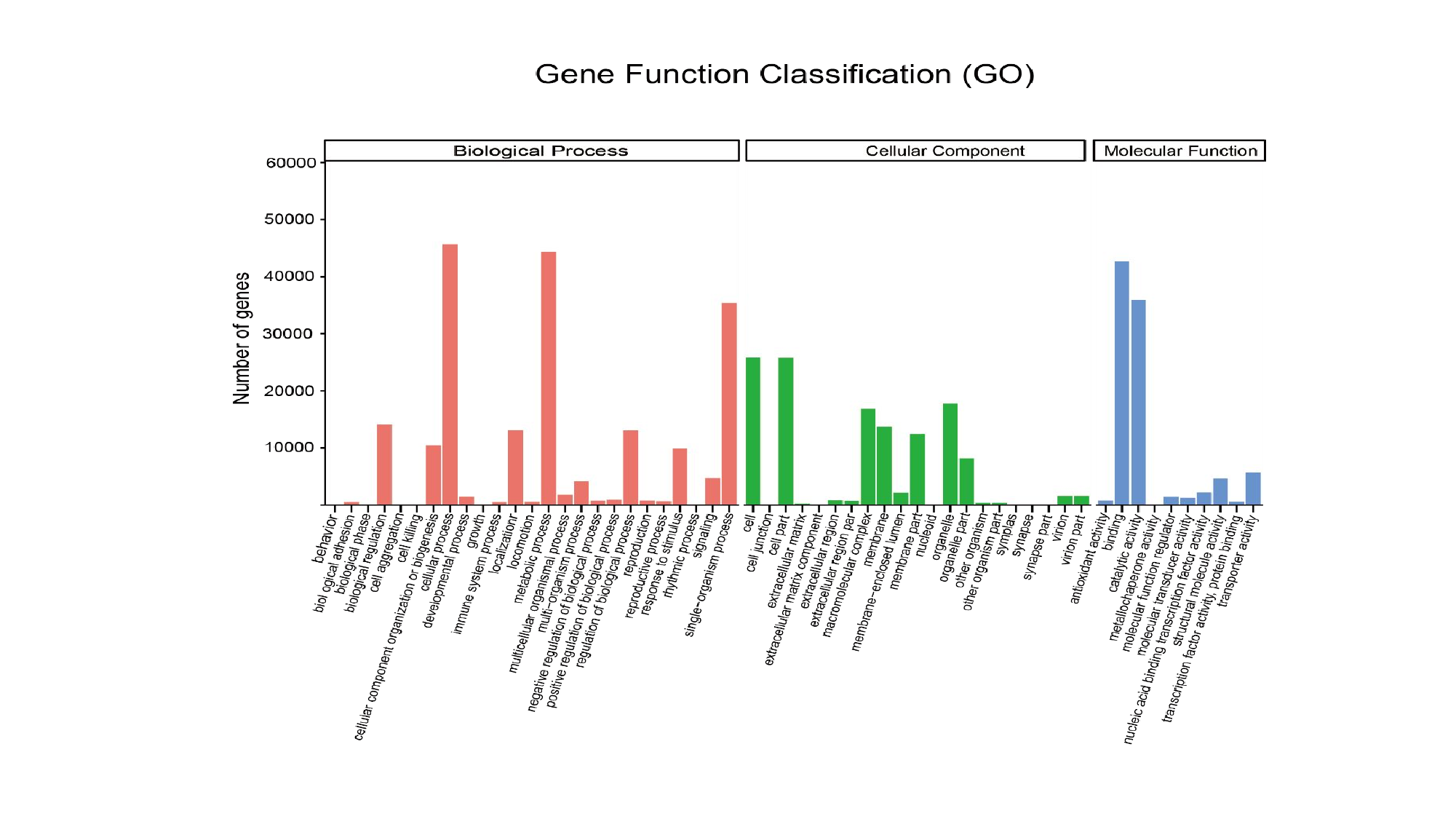

Supplement: Supplementary file 6 — Additional file 6: Figure S4. Gene Ontology (GO) classification of unigenes of A. grossedentata. [file 12870_2020_2324_MOESM6_ESM.pptx]

## Slide 1
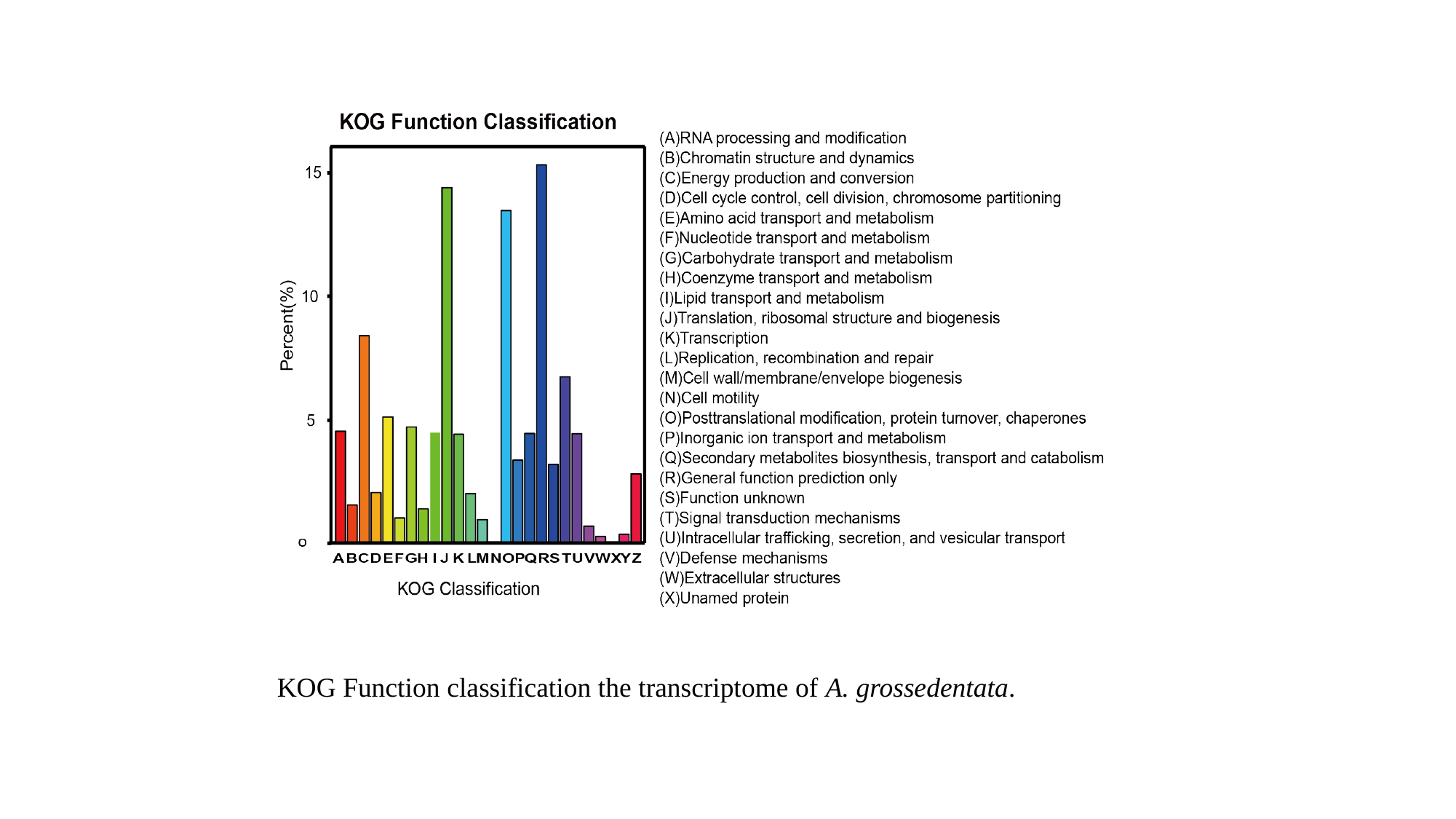

KOG Function classification the transcriptome of A. grossedentata.

Supplement: Supplementary file 7 — Additional file 7: Figure S5. KOG Function classification the transcriptome of A. grossedentata. [file 12870_2020_2324_MOESM7_ESM.pptx]

## Slide 1
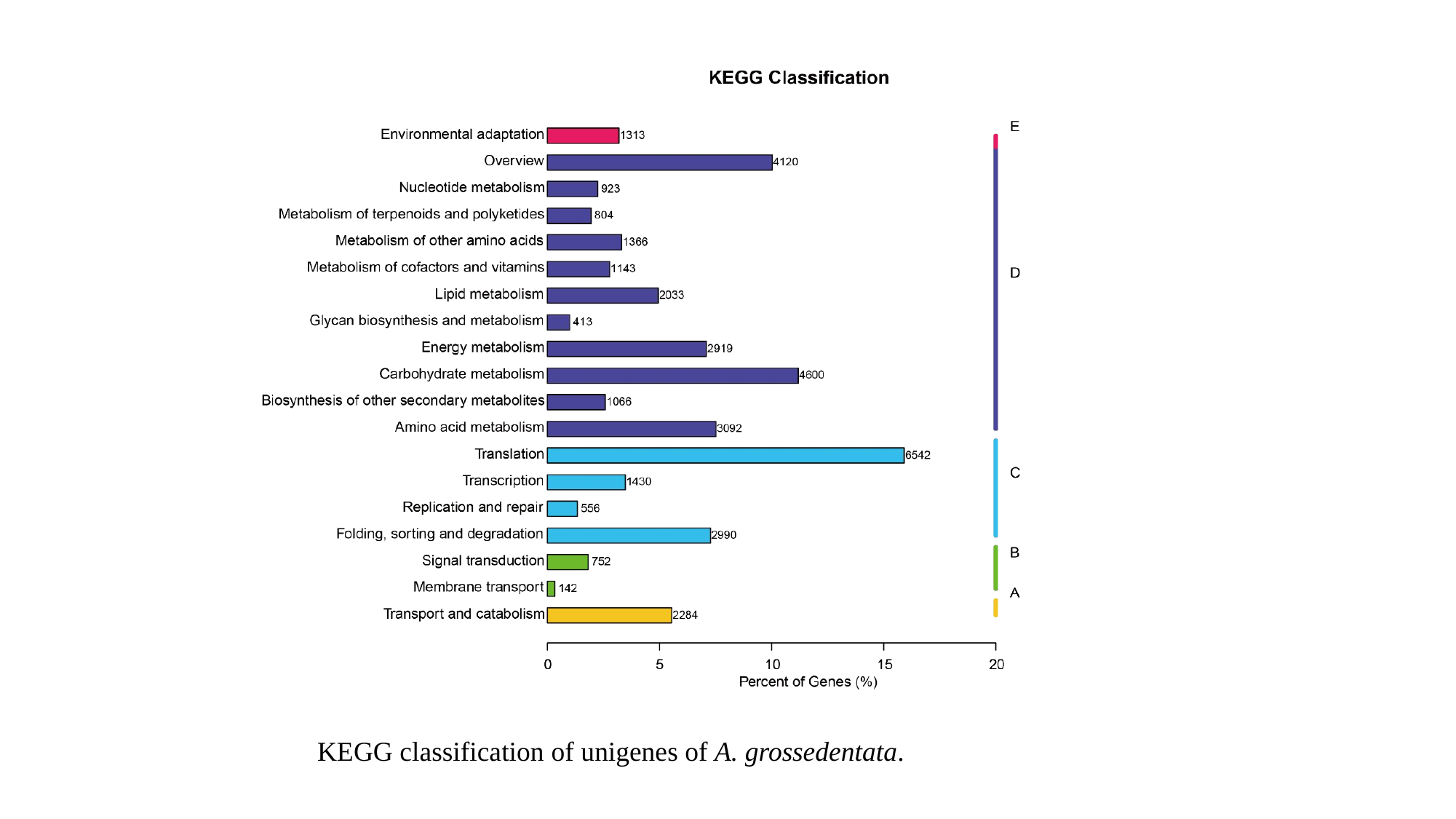

KEGG classification of unigenes of A. grossedentata.

Supplement: Supplementary file 8 — Additional file 8: Figure S6. KEGG classification of unigenes of A. grossedentata. [file 12870_2020_2324_MOESM8_ESM.pptx]
